# Stress Signaling in Cyanobacteria: A Mechanistic Overview

**DOI:** 10.3390/life10120312

**Published:** 2020-11-26

**Authors:** Raphaël Rachedi, Maryline Foglino, Amel Latifi

**Affiliations:** CNRS, Laboratoire de Chimie Bactérienne LCB, Aix Marseille University, IMM, 13009 Marseille, France; rrachedi@imm.cnrs.fr (R.R.); foglino@imm.cnrs.fr (M.F.)

**Keywords:** cyanobacteria, gene expression, signaling, stress

## Abstract

Cyanobacteria are highly diverse, widely distributed photosynthetic bacteria inhabiting various environments ranging from deserts to the cryosphere. Throughout this range of niches, they have to cope with various stresses and kinds of deprivation which threaten their growth and viability. In order to adapt to these stresses and survive, they have developed several global adaptive responses which modulate the patterns of gene expression and the cellular functions at work. Sigma factors, two-component systems, transcriptional regulators and small regulatory RNAs acting either separately or collectively, for example, induce appropriate cyanobacterial stress responses. The aim of this review is to summarize our current knowledge about the diversity of the sensors and regulators involved in the perception and transduction of light, oxidative and thermal stresses, and nutrient starvation responses. The studies discussed here point to the fact that various stresses affecting the photosynthetic capacity are transduced by common mechanisms.

## 1. Introduction

The domain of bacteria includes an ancient, monophyletic phylum of organisms called cyanobacteria which are able to undergo oxygenic photosynthesis. Their phototrophic metabolism makes them leading players in the biosphere because of their impact on the global carbon and nitrogen cycles—they are thought to account for 25% of the global primary production [1,2], and in view of the N_2_-fixing ability of some strains, they are held to be the main source of combined nitrogen in the marine environment [3]. They therefore play an important role in the fields of agriculture, aquatic ecology and environmental protection [4]. In addition, due to the great progress made in the field of genetic manipulations and the recent emergence of synthetic biology, cyanobacteria are now being applied successfully in many biotechnological processes such as bioremediation, high-value secondary metabolite synthesis, and biofuel (including ethanol and hydrogen) production [4,5]. From their early emergence up to the present day, cyanobacteria have succeeded in colonizing a wide range of aquatic to terrestrial ecological niches [6]. This impressively wide pattern of distribution is due to their ability to cope with many kinds of starvation and stress, such as nutrient deprivation, light and temperature fluctuations, and oxidative, thermal and osmotic stresses. In response to environmental changes of various kinds, the ability to trigger and coordinate suitable adaptive mechanisms depends on the ability of these bacteria to rapidly sense the physical stimuli present and to appropriately transduce the signals perceived into gene expression modulation processes.

The molecular mechanisms developed by cyanobacteria for adapting to stress conditions have been studied in detail, in several strains. In addition to the publications included in this Special Issue, these aspects have been addressed in the following reviews [7,8,9,10,11]. The aim of the present review is to sum up the latest knowledge available on the perception of and regulatory mechanisms involved in stress transduction in cyanobacteria. DNA microarray technology, and proteome analysis combined with systematic mutagenesis, has made it possible to identify several regulators involved in the stress response of the unicellular cyanobacterium model *Synechocystis* PCC 6803 (called *Synechocystis* hereafter), as reviewed in [12]. Here, we give an update of this knowledge and summarize the recent progress made in studies on stress regulation in *Synechocystis* and other cyanobacterial strains.

## 2. Light Stress

Since cyanobacteria use solar energy for their growth, the perception of light and the physiological changes that occur in response to light variations are important adaptative responses that they have to orchestrate. They are equipped with light-absorbing antenna called phycobilisomes, which are part of the main systems of acclimation to light [7]. In particular, several strains are able to vary the composition of their phycobilisomes to respond to changes in the quality of light, based on a mechanism known as complementary chromatic acclimation (CCA) [13,14]. In addition, motile strains use a process of phototaxis to move towards the optimal light conditions required for their photosynthesis [15]. CCA and phototaxis are advantageous adaptative responses that do not induce any general stress responses. They will therefore not be discussed here.

**Figure 1:**
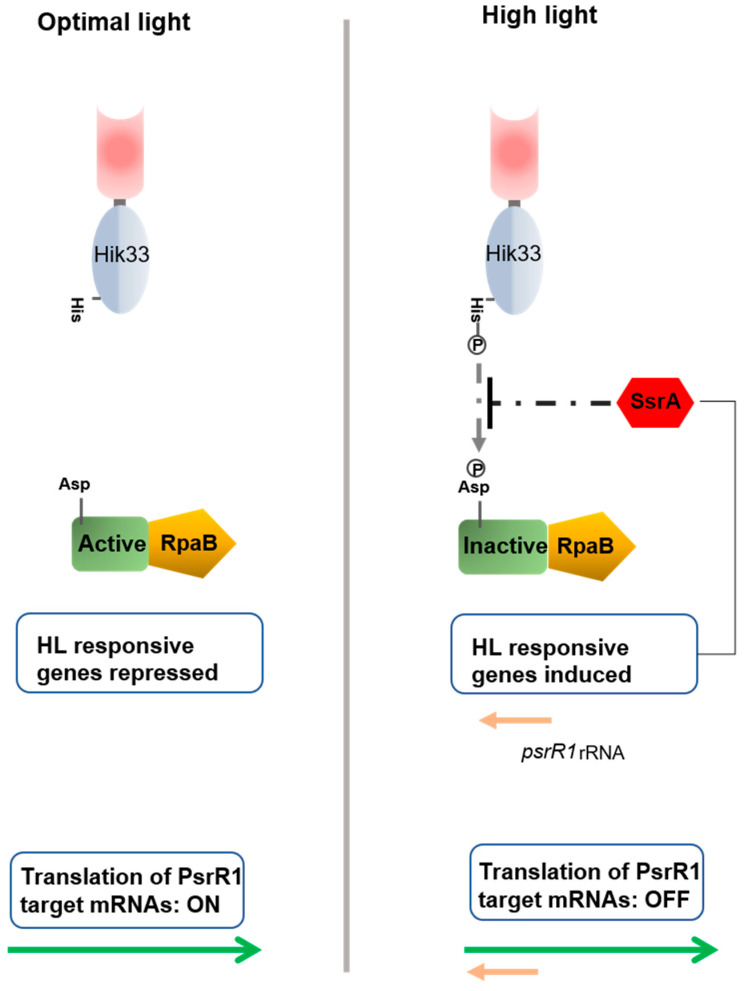
Regulation of high light stress acclimation.

In addition to qualitative changes in light, cyanobacteria can also be exposed in their environment to quantitative daily changes. Irradiances far above the light saturation level of the photosynthetic machinery are harmful, as they induce the photoinhibition and photo destruction of the photosystems [16]. In addition, when challenged by high light (HL) stress, photosynthetic organisms generate reactive oxygen species which are deleterious to all the macromolecules in the organism (see below). The adaptive responses to HL and oxidative stresses are therefore tightly bound together, which makes it difficult to identify the signal transduction systems specific to each type of condition. Short-term acclimation to strong light can be achieved by quenching excess light energy and redistributing between the photosystems the excitation energy required [17]. By contrast, long-term adaptation often requires a particular process of modulation of the patterns of gene expression. The transcription of a number of genes is induced in response to HL stress, and the promoters of several of these genes harbor a conserved tandem sequence known as the HL regulatory region (HLRR) [18,19,20]. The use of this sequence in DNA/protein interaction assays has been a decisive step towards identifying the regulatory mechanisms involved in the transmission of light stress. This is how the RpaB protein was found to be the master HL stress regulator in *Synechocystis* PCC 6803 and *Synechococcus elongatous* PCC 7942 (called *Synechococcus* hereafter) [20,21,22,23]. The *rpaB* gene, which is largely conserved in cyanobacterial genomes, encodes a response regulator protein belonging to the two-component systems. Genetic and biochemical experiments have shown that RpaB acts as a repressor of HL-induced genes during growth under normal light [24,25,26]. In *Synechococcus*, the histidine kinase NblS has been found to be the sensor partner of RpaB in the HL transduction signal [24]. NblS and its orthologue Hik33 (DpsA) in *Synechocytis* are the most highly conserved histidine kinases in the genomes of cyanobacteria [27]. The role of NblS/Hik33 in the response to HL was actually established long before that of RpaB [28,29], but establishing the proof of its direct involvement in this response has been a rather tedious task due to the fact that this kinase is a pleiotropic regulator involved in the transduction of multiple stresses (see below). In *Synechococcus*, a second response regulator (SsrA) is phosphorylated by NblS. *ssrA* gene expression is induced by HL under the control of RpaB. Once it has been produced, SsrA might quench the phosphotransfer from NblS to RpaB [24,26]. In addition, the kinase activities of NblS and Hik33 have been found to be stimulated through their interactions with SipA (NblS-interacting protein A) [30]. Since the *sipA* gene is also conserved in cyanobacterial genomes, the data derived from *Synechocystis* and *Synechococcus* might well apply to other strains. The NblS pathway involved in HL control is therefore a hierarchical cascade where two interfering response regulators (RpaB and SipA) cooperate, and in which the activity of the sensor is finely tuned to ensure optimal acclimation to HL in cyanobacteria (Figure 1).

Multiple *rpoD* genes encoding sigma factors have been identified in the genomes of cyanobacteria, and it has been suggested that many of them may act only under specific growth conditions [31]. In *Synechococcus*, the expression of the *rpoD3* gene is induced in response to HL stress under the control of RpaB, and the *rpoD3* deletion mutant is unable to survive this type of stress [23]. RpoD3 (as well as its orthologues in other cyanobacteria) might therefore be the specific alternative sigma factor enabling the polymerase to transcribe HL-induced genes.

In addition to the control process exerted at the initiation of transcription, described above, the regulation of gene expression in response to HL also occurs at the post-transcriptional level. In *Synechocystis,* a small regulatory RNA (sRNA) called PsrR1 (photosynthesis regulatory RNA1), which is conserved in the cyanobacterial genomes, has been found to be expressed in response to HL stress under the control of RpaB [32]. Based on computational and genetic findings, it has been established that PsrR1 regulates several photosynthetic genes negatively by interacting with their ribosome binding sites, and thus inhibiting the translation of their transcripts [33]. The existence of this negative regulation process is consistent with the fact that phycobilisome and photosystem reduction is one of the main physiological processes of adaptation to HL [9]. A second sRNA (RblR) accumulates under HL conditions in *Synechocystis*. RblR acts as an anti-sense RNA to the *rbcL* mRNA encoding the large subunit of the Rubisco [34]. The idea that this RNA may enhance carbon assimilation has been suggested, based on the phenotypes of mutant strains over- or under-expressing the *rblR* gene [34], but how exactly the regulation of the *rblR* transcription process induced in response to HL stress is achieved has not yet been established. In the marine cyanobacterium *Synechococcus* sp. WH7803, the expression of six non-coding RNA genes is regulated in response to HL, several of which possess photosynthesis genes as predictive targets [35]. All these data provide strong evidence that non-coding RNAs play an important role in the regulation of genes involved in the HL stress response. Elucidating the identity of all the non-coding RNA-target genes, the molecular mechanisms involved and how they are integrated into the global process of acclimation to HL is certainly the most challenging perspective ahead of us in this field.

The response regulator RpaB represses gene transcription under normal light conditions. In response to high light (HL) stress, RpaB is phosphorylated, resulting in its inactivation and the subsequent induction of HL-inducible genes. The Hik33/NblS (DspA) kinase is involved in the perception of HL stress. The putative phosphorylation of RpaB by Hik33 is thought to be inhibited by the SsrA protein. The *psrR1* gene belonging to the RpaB regulon expresses an sRNA that inhibits the translation of several genes under HL stress conditions.

## 3. Oxidative Stress

Reactive oxygen species (ROS), such as the singlet oxygen species (^1^O_2_), the hydroxyl radical (OH^•^), the superoxide anion (O_2_^−^) and hydrogen peroxide (H_2_O_2_), are by-products of aerobic metabolism, and in photosynthetic organisms they are mainly produced when the intensity of the light collected by the photosystems is greater than the rate of electron consumption [36]. Cyanobacteria, like all living organisms, have developed several defense systems which reduce and eliminate some of these ROS before they can react with biomolecules and oxidize them. The state of imbalance where the level of ROS exceeds the amount of antioxidant molecules is called oxidative stress [8,36,37]. No enzymatic defense mechanisms exist for ^1^O_2_ and OH^•^, and so the cellular responses to these species do not result in any modulation of gene expression. The regulation of oxidative stress therefore consists of the transduction of superoxide and peroxide signals.

**Figure 2:**
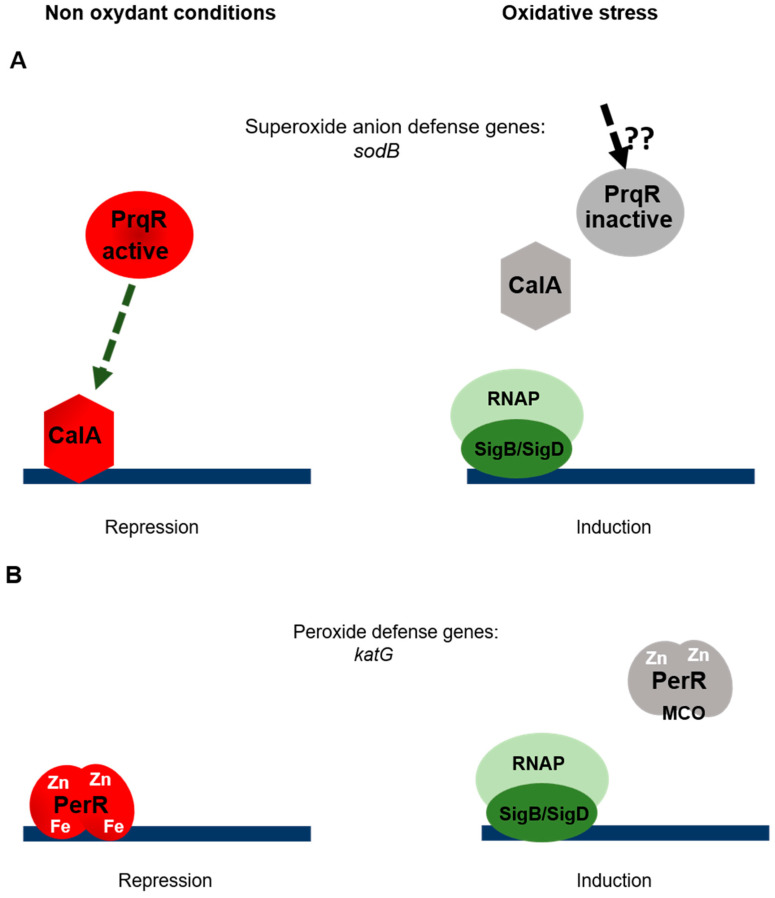
Regulation of oxidative stress responses. (**A**)Superoxide anion stress. In the absence of stress, the PrqR sensor might phosphorylate the response regulator CalA, which therefore represses the gene transcription process. In the presence of superoxide, PrqR is inactivated by a hitherto unknown mechanism. The Group 2 sigma factors SigB and/or SigD associate(s) with the RNA polymerase, resulting in the initiation of the transcription process. (**B**) Peroxide stress. The metalloregulator PerR represses gene transcription in the absence of peroxide. Under oxidative conditions, metal catalyzed oxidation (MCO) inactivates PerR, thus inducing gene transcription.

Superoxide anions resulting from the photoreduction of oxygen can be converted into H_2_O_2_ by the metalloenzymes superoxide dismutases (SOD), which are thought to constitute the main antioxidant defense system against O_2_^−^ [37]. In the multicellular cyanobacterium *Nostoc* (*Anabaena*) PCC 7120, the transcription of the *sodB* gene encoding FeSOD is under the direct negative control of the transcriptional factor CalA (cyanobacterial AbrB like) [38]. AbrB homologues, which are present in all the cyanobacterial genomes [39], have been found to regulate several metabolic pathways, such as the carbon fixation, nitrogen uptake and hydrogen oxidation pathways [40,41,42,43,44]. Since CalA is essential in *Nostoc*, obtaining a strain deleted from *calA* is not possible. The negative effect on the transcription of *sodB* was therefore analyzed by overexpressing the *calA* gene [38]. The question as to how CalA perceives the superoxide anion has not yet been answered. Interestingly, in *Synechocytis*, the transcription of the *sodB* gene was reported to be repressed by the transcriptional factor PrqR, and this control was found to be indirect [45]. If CalA also represses the transcription of *sodB* in *Synechocystis*, the regulatory scheme responsible for superoxide signaling may be based on the fact that PrqR perceives the signal and transduces it via CalA (Figure 2A).

Several studies have converged in designating PerR as the main specific regulator of the response to peroxide in cyanobacteria [46,47,48,49]. PerR is a zinc-metalloprotein, in which either Fe^2+^ or Mn^2+^ ligated to two conserved His and Asp residues act as corepressors. The activity of PerR is regulated by a metal-catalyzed oxidation (MCO) process—the metal present in the catalytic center reacts with peroxide in a Fenton-type chemistry process which leads to the irreversible inactivation of the repressor and the induction of the target genes, including peroxidase and catalase encoding genes [50]. Based on structural modeling studies, it has been suggested that the binding of PerR to its target genes may occur via multimers of PerR-protein interacting with the AT-rich repeats present in the promoters of the repressed genes [51]. The involvement of PerR in the repression of the transcription of peroxide-related genes in cyanobacteria has been described in *Synechocystis* and *Nostoc,* and metal oxidation has been found to contribute to the action of PerR in *Nostoc* [46]. In addition, the overexpression of PerR has been found to affect the composition and the stability of the photosynthetic machinery and the division process in *Nostoc* [52]. This link between PerR, photosynthesis and cell division might explain why *perR* is an essential gene in this strain.

In addition to the regulatory effects mentioned above, the response of *Synechocytis* to oxidative stress resulting from HL or peroxide treatment has also been found to rely on Group 2 alternative sigma factors, namely SigB and SigD factors [53]. A strain lacking all the Group 2 sigma factors was unable to sustain its growth when challenged by oxidative stress, although this ability was rescued by the introduction of the *sigB* or *sigD* gene. In addition, RNA polymerase holoenzyme associated with either SigB or SigD accumulates in response to peroxide stress [53]. The signaling of oxidative stress is therefore based on transcriptional regulators (CalA, PrqR, PerR) combined with the programming of the RNA polymerase with dedicated alternative sigma factors (Figure 2B).

## 4. Salt Stress

Salinity, defined as the total inorganic ion concentration in the environment, is one of the main changing abiotic factors that cyanobacteria have to cope with in both aquatic and terrestrial niches [10,54]. A high ion concentration in the medium results in an osmotic loss of water and a concomitant increase in some inorganic ions which can be deleterious to the macromolecules in the cell. By contrast, at low salinity levels, water largely flows into the cell, resulting in lysis due to high turgor pressure. Even if the acclimation to changing salinity levels is of two kinds, depending on the salt concentration in the surrounding medium, salt stress nomenclature is often attributed to high salinity conditions. Cyanobacteria, like many other non-halophilic prokaryotes, respond to salt stress by accumulating small organic solutes (often in the form of sugars) and monitoring the active export of inorganic ions. This strategy has therefore been called “the salt-out-strategy” [55]. The organic solutes in question have a low molecular mass and do not interfere with the cell metabolism, which explains why they are referred to as compatible solutes. In cyanobacteria, the strain-specific salt tolerance level is correlated with the nature of the main compatible solute produced [55].

**Figure 3:**
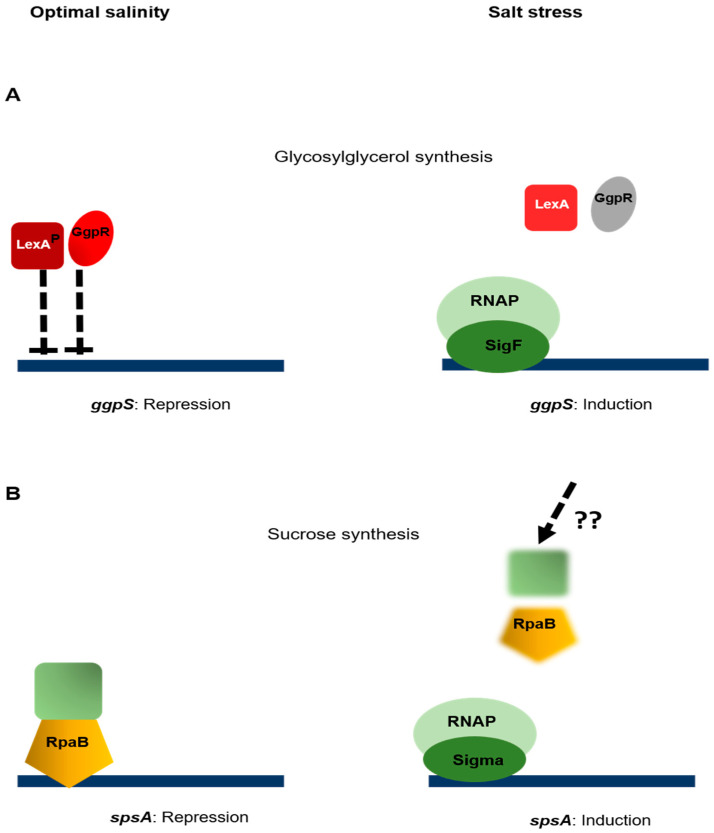
Regulation of salt stress responses. (**A**) the transcription of the *spsA* gene involved in the synthesis of sucrose is repressed by RpaB. The cognate sensor involved has not yet been identified. (**B**) the transcription of the *ggpS* gene involved in the synthesis of glycosylglycerol is subjected to the negative control of GgpR and LexA. Under salt stress conditions, the presence of the SigF sigma factor enables the RNA polymerase to initiate the transcription process. The dephosphorylation of LexA inhibits its action. The mechanism underlying GgpR inhibition is not known.

Many studies in which it was proposed to elucidate how *Synechocystis* adapt to salt stress (reviewed in [10,56,57]) have shown that the cellular responses involved are dynamic processes, and that they occur at various levels, including the allosteric regulation of the activity of several transporters and enzymes, as well as a global change in the process of gene transcription, in which the molecular mechanisms involved are not totally known. The exception here is the regulation of the transcription of genes involved in the synthesis of the organic solutes produced by *Synechocystis*, namely sucrose and glycosylglycerol. The synthesis of sucrose involves two enzymes: the sucrose phosphate phosphatase (Spp) and the sucrose phosphate synthase (SpsA), which is the rate limiting enzyme [58]. Upon exposure to salt stress, the activity of the SpsA enzyme and the transcription level of the *spsA* gene are both enhanced [59]. Under normal salinity conditions, the expression of *spsA* is repressed by the Rre39 response regulation [57], but since this is an orphan regulator (i.e., the cognate histidine kinase has not yet been identified), the question as to how the “high salinity” signal is transduced to Rre39 has not yet been elucidated. The synthesis of glycosylglycerol is a two-step reaction involving the glycosylglycerol phosphate synthase (GgpS) and the glycosylglycerol phosphate phosphatase (GgpP) enzymes [60]. Under salt stress conditions, the production of glycosylglycerol is enhanced by the induction of *ggpS* gene transcription and by an increase in the activity of the GgpS enzyme [60]. The Group 2 sigma factor SigF seems to be the specific sigma factor responsible for *ggpS* transcription, as a *sigF* mutant is unable to adapt to salt stress and shows significantly low accumulation rates of *ggpS* transcripts [61,62]. Upstream of the *ggpS* gene, the small regulatory gene *ggpR* has been identified, the deletion of which increases the *ggpS* mRNA levels under normal salinity conditions, which suggests that this gene may act as a repressor of *ggpS* expression [63]. As the GgpR protein does not contain any typical DNA binding motifs, it is questionable whether GgpR acts after binding directly to the *ggpS* promoter. The *ggpR* gene is not conserved in any other cyanobacterial genomes, but interestingly, the synteny between *ggpS* and another small gene is observed in many genomes, which means that the possible involvement of the corresponding protein in the regulation of *ggpS* expression cannot be ruled out [63]. In addition to this specific regulation, the expression of the *ggpS* gene has been shown to be controlled by the global transcriptional regulator LexA in *Synechocystis*—RNA seq analyses have shown that the transcript level of the *ggpS* gene is more than 10-fold higher in a strain lacking the *lexA* gene compared to the wild type strain, which suggests a negative control of LexA upon *ggpS* transcription. This control is likely direct, as a gel shift approach has indicated that LexA is able to interact with the promoter of *ggpS* [64]. The LexA protein has recently been proposed to be dephosphorylated on a Serine residue in response to salt stress [65]. This result might suggest that LexA represses the transcription of salt-induced genes under repressive conditions, and that their induction in response to salt stress requires the modulation of LexA activity together with the action of GgpR (Figure 3A).

Sucrose is the only osmolyte produced in *Nostoc* in response to salt stress, and in addition to being a compatible solute, it is involved in the nitrogen fixation process [66,67]. The synthesis of sucrose involves two sucrose phosphate synthases (SpsA and SpsB) and a sucrose phosphate phosphatase (SppA) [68]. Sucrose can also be formed by the reversible action of the synthases SusA and SusB, but as they are thought to preferentially catalyze the degradation of sucrose in vivo, they will not be further discussed here [66]. The increase observed in the sucrose accumulation rates in response to salt is due to higher Spps activity and the induction of the transcription of the *spsA* gene [69]. Salt induction of *spsA* was abolished in a mutant strain lacking the response regulator OrrA, which indicates that this transcriptional regulator may play a positive role in the process of sucrose synthesis [70]. The *orrA* gene has been detected in a genetic screen set up for the identification of salt-induced genes in *Nostoc* [71]; the signaling pathway leading from salt perception to OrrA activation still remains to be determined (Figure 3B).

In conclusion, the dynamics of compatible solute accumulation and the regulation of the enzymatic activities involved have both been thoroughly documented in cyanobacteria, but this is far from being the case as far as the regulatory mechanisms involved in gene expression are concerned. Since the nature of the main solute(s) produced and the ability to adapt to high salt levels vary among cyanobacteria, the possibility cannot be ruled out that the regulatory mechanisms involved may also differ from one strain to another.

## 5. Heat Shock

The optimal growth temperatures for cyanobacteria cover a large range, as this phylum includes several strains inhabiting extreme environments, from hotspring to cryosphere environments [72]. However, with the exception of these extremophile members, most mesophilic strains are sensitive to temperature fluctuations, and the processes of photosynthesis and nitrogen fixation are both inhibited by heat [73,74]; the molecular responses to heat shock are therefore crucial. Like many other organisms, cyanobacteria induce the expression of heat shock genes (*hsp*) in response to temperature upshifts [74,75,76,77]. Many of the HSPs are molecular chaperones or proteases playing a major role in proteostasis, such as the Hsp60 members (mainly GroEL and GroES), which are the most abundant HSPs produced in cyanobacteria after the occurrence of temperature upshifts.

In many bacteria, the *hsp* gene promoters contain a highly conserved 9-bp inverted repeat sequence, which is required for the heat-induction process [78]. This sequence, which has been called the controlling inverted repeat of chaperone expression (CIRCE), is the binding site of the HrcA repressor, which is also highly conserved in many bacteria [79]. HrcA is a dimeric transcriptional regulator that undergoes denaturation upon being subjected to temperature upshifts. The subsequent synthesis of GroEL regenerates the HrcA dimer, thus restoring the repression of the *hsps* genes. Chaperone activity therefore acts as the molecular heat shock sensor [80]. The *hrcA* gene is widely conserved in the cyanobacterial genomes, and its role in the repression of *hsps* genes expression has been documented in both *Synechocystis* and *Nostoc* [74,75,81]. In both strains, the deletion of the *hrcA* gene results in the constitutive expression of *hsps* genes, which is consistent with the idea that a negative control may be exerted by HrcA on heat shock genes. Microarray studies, in which the global gene response of a *Synechocystis* mutant strain deleted from the *hrcA* gene was compared with that of the wild type strain, have shown that HrcA might also regulate some genes devoid of the CIRCE element that do not belong to the Hsp60 family [75,81], which suggests that HrcA might be a global regulator of gene expression, or alternatively that the latter effect might be due to the constitutive expression of the *groEL* and *groES* genes in this mutant. Interestingly, in both *Synechocystis* and *Nostoc*, the expression of some *hsps* genes in the *hrcA*-deleted strain was not fully derepressed, since a small induction was still observed in response to a temperature upshift, which indicates that their expression is regulated by another mechanism in addition to HrcA [74,75].

A fast response to temperature upshifts is ensured by the mechanism controlling the translation of some *hsps* mRNA. This mechanism involves specific sequences located in the 5′ untranslated region of the mRNA, which change their conformation in response to heat shock [82,83]. At low temperatures, the secondary structure they generate encompasses the ribosome binding site, which affects the translation efficiency. These riboswiches, which are known as thermometer RNAs (or thermosensor RNAs), are present in many bacteria [82,83], including cyanobacteria. The *hsp17* gene of *Synechocytis* harbors a rather small 5′ untranslated region which has been found to act like a typical thermosensor [82,84], and similar cis-acting riboregulatory RNAs have been identified in *hsp* genes in *Anabaena variabilis*, *Nostoc* and the thermophilic cyanobacterium *Thermosynechococcus elongatus* [85].

**Figure 4:**
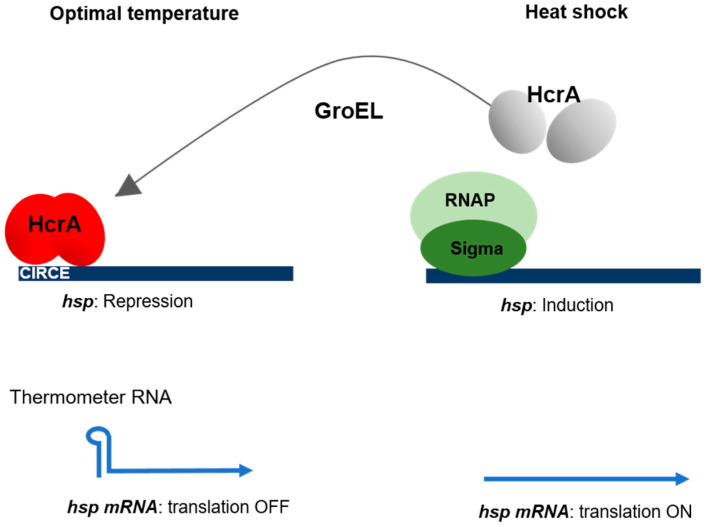
Heat shock response regulation. Under optimal growth conditions, the transcription of the *hsp* genes is repressed by HrcA, which binds to a conserved sequence called CIRCE. Upon undergoing heat shock, HrcA is inactivated and *hsp* transcription is induced. The GroEL chaperone facilitates the refolding of HrcA at the release of the stress, thus acting as the molecular sensor of the signal. Some of the *hsp* mRNAs carry an untranslated sequence forming a secondary (thermometer) structure sequestering the ribosome binding site under normal growth temperature conditions. Upon undergoing heat shock, this secondary structure dissociates, which makes it possible for the translation process to proceed.

Another regulation system of the *hsp* genes which occurs in response to heat shock consists of reprogramming the RNA polymerase core enzyme with the appropriate alternative sigma factor. In *Synechocystis*, the sigma factors SigB and SigD play an important role in high-temperature responses; the growth of a double *sigBsigD* mutant is much more severely impaired at 43 degrees than that of the simple mutants [75,86]. Interestingly, a protein (SinA) interacting with the principal sigma factor (RpoD1) and playing a role in heat shock responses has been recently identified in *Synechococcus* [87]. The RNA polymerase–RpoD–SinA complex was dissociated after a temperature upshift, a *sinA*-deletion mutant was unable to sustain its growth at 40 °C, and the induction profile of *hspA* gene was affected in the mutant. All in all, these data point to the conclusion that SinA may play a role in the replacement of RpoD1 by the heat stress-specific sigma factor. The finding that homologs of SinA were present in 361 genomes out of the 367 analyzed suggests that the function of this protein may be widely conserved among the members of the cyanobacterial phylum [87] (Figure 4).

## 6. Cold Stress

Mesophilic bacteria are often challenged by temperature downshifts to below their optimum growth temperature, which results in a decrease in the fluidity of the cell membranes and in the efficiency of the transcription and translation processes due to the abnormal stabilization of secondary structures in the DNA and RNA [88] The activities of the ribosomes and those of protein foldases are also impaired [88]. Bacteria respond by inducing the production of proteins called CSPs (cold shock-induced proteins) that serve to enhance the transcription and translation processes by acting on secondary nucleic structures; these are RNA binding proteins which affect the transcription and translation processes at low temperature via their RNA chaperoning function, and RNA helicases which stimulate the degradation or translation of RNA at low temperatures [88]. The second main response is the induction of desaturated fatty acids which counterbalance the loss of membrane fluidity [88]. The expression of the *csps* genes, which has been most closely studied in *Escherichia coli*, is regulated at the transcription, mRNA stabilization, and translation levels [89]. In *Nostoc*, the RNA-helicase encoding gene *chrC* is specifically induced in response to temperature downshifts, and its regulation was found to occur at several levels, including the transcription, mRNA stability and translation levels, but the exact molecular mechanisms involved have not yet been determined [90]. The ribosomal protein S2 has also been found to be continuously phosphorylated in *Nostoc* during exposure to cold stress, resulting in the downregulation of the translation process, with the exception of cold stress-induced mRNA [91]. In *Synechocystis*, the expression of about half of the cold-induced genes is controlled by the transmembrane histidine kinase Hik33 [92], and depends on the fluidity of the membrane [93]. Hik33 kinase also controls the responses to oxidative, osmotic and salt stress (see below), which suggests the possible existence of a common signal triggering gene induction in response to various stresses. It has been suggested that the oxidation status of the quinone pool, which was found to vary depending on the membrane fluidity during cold stress exposure, may be the common response signal to stressors affecting the membrane fluidity [94].

## 7. Nutrient Starvation

Among the multiple environmental stresses that cyanobacteria encounter, nutrient depletion is often a limiting factor for their growth. Like most bacterial species, cyanobacteria do not form typical dormant spores, but are nevertheless able to survive long periods of nutrient starvation. How these starved cells manage to survive and how they resume their metabolic activities once the nutrients are available de novo is one of the most intriguing questions being addressed today (see for examples [95,96,97]).

### 7.1. Carbone Starvation

**Figure 5:**
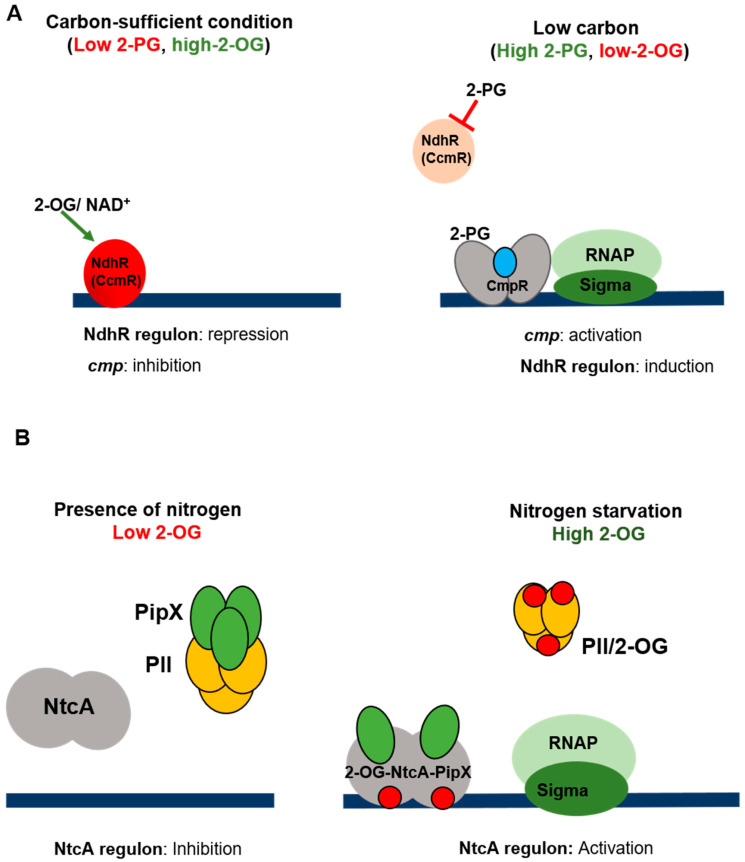
Regulation of carbon and nitrogen acclimations. (**A**) Low carbon response. NdhR (CcmR) is the main transcriptional regulator of the low carbon response. The activity of NdhR is submitted to a dual allosteric regulation where 2-OG and NAD^+^ act as co-activators and 2-PG as co-repressor. When inorganic carbon level is sufficient, the carboxylase activity of Rubisco is advantaged and 2-PG concentration is low. The levels of 2-OG and NAD^+^ are high, and NdhR is active and represses the transcription of target genes. Under carbon starvation, the oxygenase activity of Rubisco generates 2-PG that binds to NdhR and inhibits its activity, leading to the induction of CO_2_ concentrating mechanism (CCM). The *cmp* operon, which is also part of CCM, is under the positive control of CmpR. 2-PG and RuBP (1,5 ribulose biphosphate, blue dot) act as co-activators of CmpR. 2-PG and RubP are generated by the oxygenase activity of RubisCo under low carbon conditions. The low carbon response is also regulated by LexA and AbrB and submitted to post-transcriptional regulations (see text for details). (**B**) Nitrogen starvation. The NtcA protein, the main transcriptional factor of nitrogen-induced genes, is activated by PipX. The intracellular level of 2-OG (red circles), which reflects the nitrogen status of the cell, is perceived by PII and NtcA. Under replete nitrogen conditions, the 2-OG level is low, PipX interacts with PII, and NtcA is inactive. When nitrogen is a limiting factor, 2-OG accumulates, and PipX changes its partner and interacts with NtcA. The 2-OG-NtcA-PipX complex regulates the gene transcription process. The post-transcriptional regulation mechanisms involved in nitrogen starvation signaling are not presented in the figure.

Being autotrophic organisms, cyanobacteria use inorganic carbon (CO_2_ and bicarbonate) as a carbon source for their growth [2]. They are able to acclimate to limited carbon conditions by optimizing their carbon fixation activity through a mechanism that concentrates CO_2_ near the ribulose biphosphate carboxylase/oxygenase enzyme (RubisCo) [98,99]. This mechanism (known as CCM for carbon concentrating mechanism) includes carboxysomes and transporters for the internalization of dissolved inorganic carbon [100]. Two transcriptional regulators (CmpR and NdhR) belonging to the LysR family are the main specific factors ensuring gene expression modulation in response to carbon starvation [11]. CmpR has been identified in *Synechocystis,* and has been shown to activate the transcription of the *cmp* operon that encodes a bicarbonate transporter in response to low carbon conditions [101]. The activity of CmpR has been found to be stimulated by the binding of 2-phosphoglycolate (2-PG), which is generated from the oxygenation of ribulose-1,5-bisphosphate (RuBP) by the oxygenase activity of Rubisco under low CO_2_ conditions [102]. 2-PG is a toxic by-product of the RubisCO oxygenase reaction that is metabolized by the essential photorespiration process [99]. Structural studies indicated that CmpR acts as a dimeric protein with one molecule of RuBP bound between two monomers [103], but the association between CmpR and 2-PG has not been structurally demonstrated yet. In *Nostoc*, transcription of the *cmpR* gene is positively regulated in response to combined nitrogen starvation, making this regulator a factor connecting nitrogen and carbon metabolisms [104]. The involvement of NdhR (also called CcmR) in the control of the response to the low carbon condition has been established in *Synechocystis* for the first time [105]. NdhR represses the transcription of a large set of genes, including the *ndh3* operon encoding NAD(P)H dehydrogenase (*ndh*) subunits [105], the *sbtA/B* genes encoding the Na^+^/HCO_3_^−^ symporter, and genes belonging to the *ndh-I3* operon that encodes for the high-affinity CO_2_ uptake system subunits [106,107]. NdhR has also been found to regulate all the identified low-carbon-inducible genes in the coastal strain *Synechococcus* PCC 7002 [108]. The negative control of gene expression by NdhR can be thus considered as the main adaptive mechanism in carbon limitation. Like CmpR, NdhR is submitted to allosteric regulation. In vitro analysis has shown that the binding of NAD^+^ and 2-oxoglutarate (2-OG), accumulating under carbon-sufficient conditions, enhances the NdhR DNA binding activity [109]. Further progress in the understanding of the allosteric regulation of NdhR has been achieved recently [110]. A combination of in vivo and structural approaches has confirmed that the role of 2-OG acts as co-repressor, and established 2-PG as the inducer (co-repressor) as it inhibits NdhR binding to DNA [110]. 2-OG is one of the two signaling molecules of nitrogen starvation (see below), and therefore NdhR coordinates the responses to nitrogen and carbon status in cyanobacteria (Figure 5A). In addition to the control exerted by CmpR and NdhR, the AbrB-type regulator cyAbrB2 has been shown to adapt carbon and nitrogen metabolisms to the photosynthetic activity in *Synechocystis* [111], and LexA has been found to be required for surviving low carbon conditions [112].

Interestingly, the transcription of several genes expressing sRNAs is modulated according to the availability of inorganic carbon in *Synechocystis* (CsiR1, Ncr0700, NcR1200, SyR12) [107,113]. As this response has been found to be maintained in a strain lacking the *ndHR* gene, a post-transcriptional regulation acting independently from NdhR may contribute to the acclimation to carbon starvation.

In addition to CCM induction, the expression of the genes encoding the flavodiiron proteins Flv2 and Flv4, acting as electron valves to protect the photosystems from oxidation [114], is also highly induced after a shift to low carbon [115]. The transcript of the *flv4-2* gene has been shown to be negatively regulated by its anti-sens RNA As1_flv4 [115]. This control of Flv protein production extends the post-transcriptional regulation to mechanisms adjusting photosynthesis to carbon availability.

### 7.2. Nitrogen Starvation

Cyanobacteria survive prolonged nitrogen starvation by decreasing their central metabolism and by degrading their photosynthetic apparatus, resulting in a loss of autofluorescence and cell bleaching, a state called chlorosis (Reviewed in [116]). Chlorosis is a highly orchestrated process which starts with the synthesis of the Clp-protease adaptor protein NblA [117]. Since the regulation of the *nblA* gene has recently been reviewed [118], we will not discuss this topic here, but rather focus on specific signaling pathways involved in the transduction of nitrogen deficiency.

Although cyanobacteria are able to assimilate a number of combined nitrogen compounds, including ammonium, nitrate, nitrite and urea, the preferred one is ammonium [119,120,121]. The metabolism of various compounds therefore starts with their intracellular assimilation to ammonium, which is then incorporated into the carbon skeleton of 2-OG via the glutamine synthetase–glutamate synthase (GS-GOGAT) pathway, giving glutamate [122]. The fact that 2-OG is an intermediate of the TCA cycle means that the processes of nitrogen and carbon assimilation are interconnected. A state of combined nitrogen deficiency therefore leads to inhibition of the GS-GOGAT cycle, and ultimately to 2-OG accumulation, which provides us with a useful indicator of the nitrogen status of the cell [119].

Non-diazotrophic cyanobacterial strains have to cope with nitrogen deficiency, and this adaptative response depends on the ability to perceive the state of starvation and to modulate the pattern of gene expression accordingly in order to use alternative nitrogen sources. The facultatively nitrogen-fixing cyanobacteria also have to perceive the state of combined nitrogen depletion in order to induce the genetic program enabling them to shift their metabolism towards the reduction of atmospheric nitrogen. In both cases, 2-OG has been found to act as a molecular sensor of nitrogen deficiency [120]. We will focus below on the response to nitrogen starvation in non-fixing cyanobacteria, as it is only in these organisms that this situation constitutes a stress [116]. The transduction of the 2-OG signal, which has been intensively studied in unicellular freshwater strains (mainly *Synechocystis* and *Synechococcus*), involves several factors, including the transcriptional regulator NtcA, which is thought to be the main one involved. The NtcA protein is a member of the CRP family of transcription regulators, and deletion of the *ntcA* gene impairs the ability of the strains to grow on nitrogen sources other than ammonium, which is consistent with the finding that NtcA activates the transcription of genes required for the assimilation of nitrogen sources, such as nitrate [120,123]. The activity of NtcA is modulated depending on the nitrogen source and its concentration: it is induced in the absence of ammonium and under conditions where the 2-OG level is high (which corresponds to low levels of nitrogen) [124]. A dimer of NtcA has the ability to bind to 2-OG, and structural studies have established that the binding of the effector induces a conformational change that enhances the DNA-binding activity [125,126]. PipX, a protein present only in cyanobacteria, acts as a coactivator of NtcA [127]. Biochemical studies have shown that PipX enhances the affinity of NtcA for promoters and the effective affinity of NtcA for 2-OG [128]. In addition to NtcA, the second sensor at work in 2-OG signaling is the protein PII, which is encoded by the *glnB* gene present in all the cyanobacterial genomes. PII proteins constitute one of the largest and most widely distributed families of signal transduction factors, all the members of which are able to bind to ATP/ADP in addition to 2-OG (for a recent review on PII, see [129]). Via protein–protein interactions, they control the activity of target proteins in response to cellular ATP/ADP levels and the 2-OG status, thus creating a link between the carbon and nitrogen metabolisms [129]. When the combined nitrogen source is abundant (and the intracellular 2-OG level is low), PII-ADP binds to PipX and NtcA is mainly present in the apo form, whereas under low combined nitrogen levels (and high 2-OG concentrations) PII interacts with 2-OG and ATP, inhibiting its interactions with PipX. At the same time, the binding of 2-OG to NtcA favors its interaction with PipX, resulting in the enhancement of the transcriptional activation of the genes involved in nitrogen assimilation [126,127]. The role played by PII and its protein partners in the control of the nitrogen/carbon balance in cyanobacteria has been intensively discussed over the last few years. For further information on this subject, readers can consult the following reviews [121,130,131] (Figure 5B).

The regulation of nitrogen assimilation in cyanobacteria also occurs at a post-transcriptional level through two distinct mechanisms: riboswiches and sRNA. (i) Comparative genome analyses designed for riboswitch probing have identified the presence of two RNA motifs (*glnA* and Downstream peptide) present only in cyanobacterial genomes and metagenomic sequences [132]. Shortly after, these two RNA motifs have been shown to specifically bind glutamine [133]. In a study comparing 60 cyanobacterial genomes, these RNA motifs have been found to be frequently present in the gene *gifB* that encodes the IF17 protein involved in the inactivation of the glutamine synthetase [134]. In this study, it has been proposed to rename the *glnA* and Downstream peptide as glutamine type 1 and glutamine type 2 riboswiches, respectively, which will avoid the confusion between the *glnA* RNA motif and the *glnA* gene encoding glutamine synthetase. The RNA motif located at the 5′ UTR of the *gifB* of *Synechocystis* has been proven to act as a riboswich since its interaction with glutamine increased protein synthesis in vivo [134]. These data unearthed the role of glutamine as a signaling molecule, specifically in cyanobacteria. (ii) Transcriptome and ChlP seq analyses have shown that several genes expressing small regulatory RNA are responsive to nitrogen starvation [113]. The transcription of two of them (NsrR1 and NsRi4) is under the control of NtcA in *Nostoc* and *Synechocystis* [135,136,137]. In *Nostoc*, NsrR1 (nitrogen repressed RNA1) has been shown to regulate negatively the expression of a gene required for diazotrophic growth [135], and to be involved in the expression of the *nblA* gene [135], which points to a role of this RNA in the control of nitrogen metabolism and chlorosis in this bacterium. NsiR4 (nitrogen stress-induced RNA 4) has been shown to be required for the negative regulation of the *gifA* gene encoding the glutamine synthetase inactivation factor in *Synechocystis* and *Nostoc* [136]. Since genes expressing NsiR4 are widely conserved in the genomes of cyanobacteria [136], the control of nitrogen assimilation by this snRNA is likely to also be conserved.

### 7.3. Phosphate Starvation

**Figure 6:**
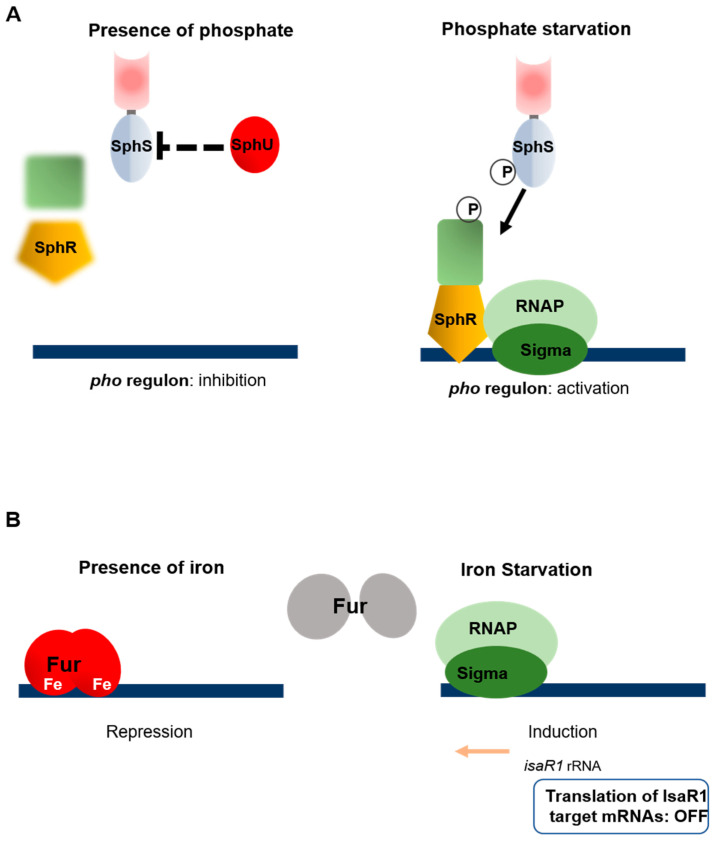
Regulation of phosphate and iron acclimations. (**A**) Phosphate starvation. The group of genes induced in response to phosphate starvation is called “the *pho* regulon”. In response to phosphate limitation, the transcription of these genes is activated by the TCS formed by the kinase SphS and the response regulator SphR. Under replete phosphate conditions, the activity of SphS is inhibited by the protein SphU, presumably by interacting with the transmitter domain of SphS. (**B**) Iron starvation. The Fur repressor is a metalloprotein in which iron serves as a cofactor. When iron becomes limited, it is inactivated by the shift of Fur to its apoform, and the transcription process is thus induced. In addition, the translation of several genes is inhibited by the sRNA isaR1, which is expressed under iron starvation conditions.

Phosphate deficiency affects photosynthetic activity, cell growth, phospholipid and nucleotide synthesis, and cell growth. When starved of phosphorus, cyanobacteria induce the expression of specific genes (known as the Pho regulon), which enhances phosphate uptake [138,139,140], triggers a process of alkaline phosphatase synthesis releasing phosphorus from several components [141,142,143], and decreasing the phospholipid levels present in the membrane via a remodeling process [144]. The Pho regulon has been found in *Synechocystis* [145,146] and *Synechococcus* [147,148] to be under the exclusive positive control of the SphS/SphR two-component system. The signal transduction mechanism performed by the SphS/SphR system has been studied in *Synechocystis*; the fact that the deletion of the extended N-terminal extremity of the SphS kinase sensor abolished the activation of the Pho regulon suggests that this sequence is required for sensing the phosphorus levels [149]. The activity of the SphS/SpHR system is negatively regulated by the SphU protein, probably by interacting with and inactivating the transmitter domain of SpHS [150]. Gel mobility shift assays have shown the existence of a conserved sequence in the promoters of genes belonging to the Pho regulon, known as the ‘Pho box’, which is required for the activation of transcription by SphR [146]. The Pho regulon has been predicted to exist in 19 cyanobacterial strains, and interestingly, the loss of SphS/ SphR was observed in the genomes of 3 of them known to inhabit phosphate-rich niches [151]. Whether the need to adapt to phosphate deficiency has been lost in the course of evolution or whether a regulator other than SphR is involved in these strains still remains to be elucidated (Figure 6A).

### 7.4. Iron Starvation

Iron plays the role of cofactor in the case of several essential proteins, but free iron is rarely available in nature, which makes it an important limiting factor for bacterial and phytoplankton growth in various environments [152]. Iron homeostasis is tightly regulated to prevent both starvation and excess, which lead to oxidative stress in cyanobacteria [8]. The ferric uptake regulator (Fur) is the main transcriptional regulator of the genes involved in iron homeostasis in most bacteria [153]. Fur acts as a repressor and an iron sensor; at higher iron concentrations, it binds to Fe^2+^, dimerizes, and binds to target promoters in a conserved sequence termed “Fur-box”. During iron starvation, the release of Fe^2+^ inactivates Fur and cancels the repression exerted by this regulator [154]. Fur homologues are widely distributed in cyanobacterial genomes, and the involvement of Fur in their adaptation to iron starvation has been investigated in some model freshwater strains (*Nostoc*, *Synechococcus, Synechocytis)* [155,156,157]. Interestingly, the latter studies have shown that the *fur* gene is essential in cyanobacteria, which suggests that it is required for some essential processes in addition to iron-response control [155,156,157]. Studies using a “transcript-depletion” strategy have shown that in *Nostoc*, Fur controls the expression of genes involved in several processes, including exopolysaccharide biosynthesis, phycobilisome degradation, chlorophyll catabolism, nitrogen fixation, and exopolysaccharide biosynthesis [158]. Several functions must be inhibited in response to iron starvation, but how could this control be exerted since Fur is inactivated under these conditions? A recent study on *Synechocytis* has yielded a clue to understanding the molecular basis of this homeostasis [159]. The gene expressing the small regulatory RNA Isar1 (Iron Stress-Activated RNA 1) is repressed by Fur. Isar1 accumulates in response to iron starvation and controls at a post-transcriptional level the expression of several genes involved in central cellular processes (photosynthesis, (iron–sulfur) cluster biogenesis, citrate cycle and tetrapyrrole biogenesis [159]. Homologs of IsaR1 are largely conserved in the genomes of cyanobacteria [159], and the involvement of this riboregulator in the control of photosynthesis via iron homeostasis might be conserved in the cyanobacterial phylum (Figure 6B).

## 8. Multiple Stresses Sharing Common Sensors and/or Transducers 

All the environmental stresses discussed above generally decrease the maximum photosynthetic capacity of cyanobacteria, resulting in the hyper-reduction of the electron flow and ultimately in a decrease in the anabolism. It is therefore often difficult to distinguish between the specific effect of a particular stress and its indirect impact through a change in the redox state of the cell. This distinction could be made in the case of nitrogen deficiency in *Synechoccocus* [160]. In this study, nitrogen deficiency was mimicked using an inhibitor of the glutamine synthetase. This treatment resulted in the induction of the *glnB* gene (specific to nitrogen deficiency), and of the *nblA* gene (chlorosis marker). Interestingly, the addition of nitrate suppressed the induction of *nblA* without significantly affecting the expression of *glnB*. By acting as an electron sink, nitrate decreased the hyper-reduction of the photosystems and probably modified the redox state, which might therefore be considered as the specific signal triggering *nblA* [160].

Genetic screening for mutations impacting the adaptive responses to several stresses at work has led to the identification of the sensor Hik33/NblS (DspA) and the cognate response regulators as leading players in the transduction of the stress signals (reviewed in [12]). Photosynthetic redox stress can be assumed to be the actual signal that is perceived by Hik33/NblS/(DspA) kinase during exposure to various stresses. As mentioned above, the redox state of the quinone pool reflects the fluidity status of the membrane [94]. The redox-sensitive transcriptional regulator PedR has been found to be reduced by thioredoxin and to be inactivated under HL conditions, which shows the existence of a relationship between gene expression and photosynthetic activity [161]. Gene regulation through PedR can thus be expected to respond to various stresses that affect the photosynthetic abilities of these bacteria. In addition to the redox signal that mediates a pleiotropic transduction pathway, more specific regulatory cross-talk occurs between some stress responses.

## 9. Conclusions

Given the ecological role of cyanobacteria, their wide pattern of distribution and their versatile metabolism, data on the stress responses at work in these bacteria are relevant to many fields, including industrial biotechnological applications. A thorough knowledge of the regulatory networks mediating stress responses is a prerequisite for circumventing inhibitory mechanisms in order to maintain the growth of these microorganisms, even under the unfavorable conditions that frequently occur during large-scale production processes.
